# The present and the future of antimicrobial resistance surveillance in Catalonia (Spain), 2024: from manual to automatic notification

**DOI:** 10.1186/s13756-026-01781-0

**Published:** 2026-06-30

**Authors:** Marc Bach-Griera, Helios Gayà, Aurora Sabrià, Luca Basile, Nieves Larrosa, Cristina Pitart, Alba Rivera, Guillem López de Egea, Yuliya Poliakova, Mónica Ballestero-Téllez, Jun Hao Wang-Wang, Antonio Casabella-Pernas, Sandra Esteban-Cucó, Frederic Gómez-Bertomeu, Esther Clapés, Alba Bellés-Bellés, Inés Valle T-Figueras, José Domenico Stavola, Isabel Pujol-Bajador, Glòria Trujillo, Anna Vilamala, Araceli González-Cuevas, Mayuli Armas-Cruz, Mariona Xercavins, Virginia Plasencia, Natàlia Roca-Bau, Manel Monsónis, Núria Torrellas-Bertran, Karolina Romero-Vanegas, Esther Sanfeliu, Ester Comellas, Gemma Flores-Mateo, Fina Guimerà, Anna Llimós-Fàbregas, Maria Font-Font, Carles Alonso-Tarrés, Jacobo Mendioroz, Pilar Ciruela

**Affiliations:** 1 Agència de Salut Pública de Catalunya, Subdirectorate General for Public Health Surveillance and Emergency Response, Barcelona, Spain; 2 Microbiology Service, Vall d’Hebron Hospital Universitari, Barcelona, Spain; 3 Microbiology Service, Hospital Clínic de Barcelona, Barcelona, Spain; 4 Microbiology Service, Hospital de la Santa Creu i Sant Pau, Barcelona, Spain; 5 Microbiology Service, Bellvitge University Hospital, L’Hospitalet de Llobregat, Spain; 6 Instituto de Salud Carlos III, Research Network for Respitary Diseases, Madrid, Spain; 7 Microbiology Service, Consorci del Laboratori Intercomarcal de l’Alt Penedès, l’Anoia i el Garraf (CLILAB), Vilafranca del Penedès, Spain; 8 Clinical Microbiology Service, CATLAB, Viladecavalls, Spain; 9 Microbiology Service, Laboratori Clínic Metropolitana Nord, Microbiology Service Hospital Universitari Germans Trias i Pujol, Badalona, Spain; 10 Department of Genetics and Microbiology, Autonomous University of Barcelona, Cerdanyola del Vallès, Spain; 11 Microbiology Service, Corporació Sanitària Parc Taulí, Sabadell, Spain; 12 Institute of Research and Innovation Parc Tauli (I3PT), Autonomous University of Barcelona, Sabadell, Spain; 13 Microbiology Service, Laboratori de Referència de Catalunya (LRC), El Prat de Llobregat, Spain; 14 Microbiology Section, Hospital Universitari Joan XXIII de Tarragona, Tarragona, Spain; 15 Centre for Biomedical Research in Infectious Diseases Network (CIBERINFEC), Instituto de Salud Carlos III, Mataró, Spain; 16 Microbiology Section, Laboratori Clínic Territorial de Girona, Lleida, Spain; 17 Microbiology and Parasitology Unit, Hospital Universitari Arnau de Vilanova, Salt, Spain; 18 Microbiology Section, Hospital de Mataró-Laboratori de Referència de Catalunya, Mataró, Spain; 19 Microbiology Section, Corporació de Salut del Maresme i la Selva- Hospital Comarcal Sant Jaume de Calella - Hospital Comarcal de Blanes, Calella, Spain; 20 Microbiology Laboratory, Clinical Analysis Service, Hospital Universitari Sant Joan de Reus - Laboratori de Referència de Catalunya, Institut d’Investigació Sanitària Pere Virgili, Reus, Spain; 21 Department of Basic Medical Sciences, Faculty of Medicine and Health Sciences, Institut d’Investigació Sanitària Pere Virgili, Rovira and Virgili University, Reus, Spain; 22 Microbiology Section, Xarxa Assistencial Universitària Althaia, Manresa, Spain; 23 Microbiology Section, Hospital Universitari de Vic, Vic, Spain; 24 Microbiology Section, Parc Sanitari Sant Joan de Déu, Sant Boi de Llobregat, Spain; 25 Microbiology Section, Fundació Privada Hospital Asil de Granollers, Granollers, Spain; 26 Microbiology Section, University Hospital Mútua de Terrassa - CATLAB, Terrassa, Spain; 27 Microbiology Department, Hospital Terrassa - CATLAB, Terrassa, Spain; 28 Clinical Microbiology Section, Laboratorio Echevarne, Esplugues de Llobregat, Microbiology Service Spain; 29 Microbiology Department, Hospital Sant Joan de Déu Barcelona, Tarragona, Spain; 30 Microbiology Service, Hospital de Palamós, Palamós, Spain; 31 Microbiology Service, Fundació Salut Empordà - Hospital de Figueres, Figueres, Spain; 32 Clinical Analysis Service, Hospital d’Olot Comarcal de la Garrotxa, Olot, Spain; 33 Microbiology Service, Salut Catalunya Central - Hospital de Berga, Berga, Spain; 34 Preventive Medicine and Epidemiology Service, Hospital Sant Pau i Santa Tecla, Tarragona, Spain; 35 Microbiology Service, SNB Diagnósticos Globales - Eurofins Clinical Diagnostics, Esplugues de Llobregat, Spain; 36 Microbiology Department, Laboratori Cerba Internacional, Sabadell, Spain; 37 Clinical Analysis Service, Hospital Comarcal del Pallars, Tremp, Spain; 38 Microbiology Department Laboratory, Puigvert Foundation, Barcelona, Spain; 39 CIBER Epidemiología y Salud Pública, Instituto de Salud Carlos III, Madrid, Spain

**Keywords:** Antimicrobial resistance, Surveillance systems, ESKAPE, Automated notification, Laboratory notification, Healthcare-associated microorganism

## Abstract

**Background:**

Antimicrobial resistance (AMR) remains a major global public health concern requiring coordinated surveillance and technological innovation. In Catalonia, AMR surveillance is conducted through the Microbiological Reporting System of Catalonia (MRSC) and will be integrated into the Epidemiological Surveillance Information System (SIVEC), enabling automated and real-time monitoring. This study aimed to evaluate the current landscape of AMR surveillance and laboratory capacity across clinical microbiology laboratories (CMLs).

**Methods:**

All CMLs participating in the MRSC were invited to complete a structured survey assessing diagnostic capacity, resistance mechanism (RM) detection, laboratory information systems (LIS), and reporting practices. Surveillance data from invasive isolates (one per patient and episode) collected between 2016 and 2023 were analysed. CMLs were classified by complexity (L1–L3) according to phenotypic, molecular and genomic capabilities. Temporal trends in resistance were assessed using logistic regression models.

**Results:**

By 2023, 53 CMLs participated compared with 28 in 2016, reflecting a marked increase in coverage. A total of 87,556 invasive isolates and 694,289 susceptibility tests were reported. *Escherichia coli* was the most frequently notified microorganism (37,999 isolates), followed by *Klebsiella pneumoniae* (12,626) and methicillin-susceptible *Staphylococcus aureus* (10,464). All laboratories followed EUCAST criteria, although only 56.6% had implemented the 2023 version. Most CMLs (92.5%) detected RMs and 73.6% monitored multidrug resistance. Based on complexity, 18.9% were L1, 66.0% L2, and 15.1% L3; most notifications were reported by L2 (57.4%) and L3 laboratories (39.4%). Carbapenem resistance decreased in *Pseudomonas aeruginosa* (21.7 to 12.9%) and *K. pneumoniae* (6.3% to 3.2%), whereas *E. coli* increased (0.07 to 0.52%). Vancomycin resistance increased in *Enterococcus faecium* (0.27 to 2.30%) and remained low in *E. faecalis*. Third-generation cephalosporin resistance increased in *E. coli* (14.4 to 17.8%) and decreased in *K. pneumoniae* (36.0 to 26.6%), while MRSA declined (33.1 to 20.6%).

**Conclusions:**

AMR surveillance in Catalonia provides robust and representative data across a heterogeneous network. Further efforts are needed to ensure consistent EUCAST implementation and expand RM detection. The implementation of SIVEC will enable integrated, real-time surveillance and support timely, evidence-based public health decision-making.

**Supplementary Information:**

The online version contains supplementary material available at https://doi.org/10.1186/s13756-026-01781-0.

## Background

Antimicrobial resistance (AMR) remains a critical global public health concern [1]. Without effective intervention, AMR could become the leading cause of death worldwide by 2050, resulting in approximately 10 million deaths annually, surpassing current global cancer mortality rates [2, 3]. This high mortality rate and its potential impact on the global economy have led the European Commission to identify AMR as one of its three main priority health threats [4]. Recently, the World Health Organization (WHO) published an updated list of priority bacterial pathogens. These pathogens have been identified as requiring particular attention in research, development, and public health interventions, due to issues of transmissibility, treatability, and prevention [5]. These list includes Gram-negative bacteria resistant to last-resort antibiotics, drug-resistant *Mycobacterium tuberculosis*, and other resistant pathogens with a high disease burden, such as *Salmonella* spp., *Shigella* spp., *Neisseria gonorrhoeae*, *Pseudomonas aeruginosa*, and *Staphylococcus aureus*.

In Catalonia, AMR surveillance must be reported to the Department of Public Health of Catalonia (Decree 203/2015 and Order SLT/205/2019) [6, 7]. The Decree establishes the Epidemiological Surveillance Network of Catalonia (XVEC) and regulates the reporting systems for notifiable diseases and epidemic outbreaks: Statutory Disease Reports system (SDR) and Microbiological Reporting System of Catalonia (MRSC). Both systems are coordinated by the Subdirectorate General for Surveillance and Response to Public Health Emergencies (SGVRESP) of the Public Health Agency of Catalonia (ASPCAT).

The MRSC collects and analyses laboratory data on confirmed notifiable infectious diseases and their AMR profile, including those microorganisms that can cause healthcare-associated infections.

This study aimed to characterise the current landscape and explore future perspectives of AMR surveillance through a comprehensive survey of clinical microbiology laboratories (CMLs) participating in the MRSC. In addition, it sought to inform the development of a clinical microbiology laboratories network by identifying key gaps and opportunities to strengthen AMR surveillance capacity. The survey included questions related to microbiological diagnostic capacity, phenotypic and genotypic techniques, and testing outsourcing practices.

Furthermore, information from the Laboratory Information System (LIS) of each CML was collected to support the organisation and future implementation of the Epidemiological Surveillance Information System of Catalonia (SIVEC), enabling a transition towards an automated and real-time surveillance model.

## Methods

### Participating centres

**Fig. 1:**
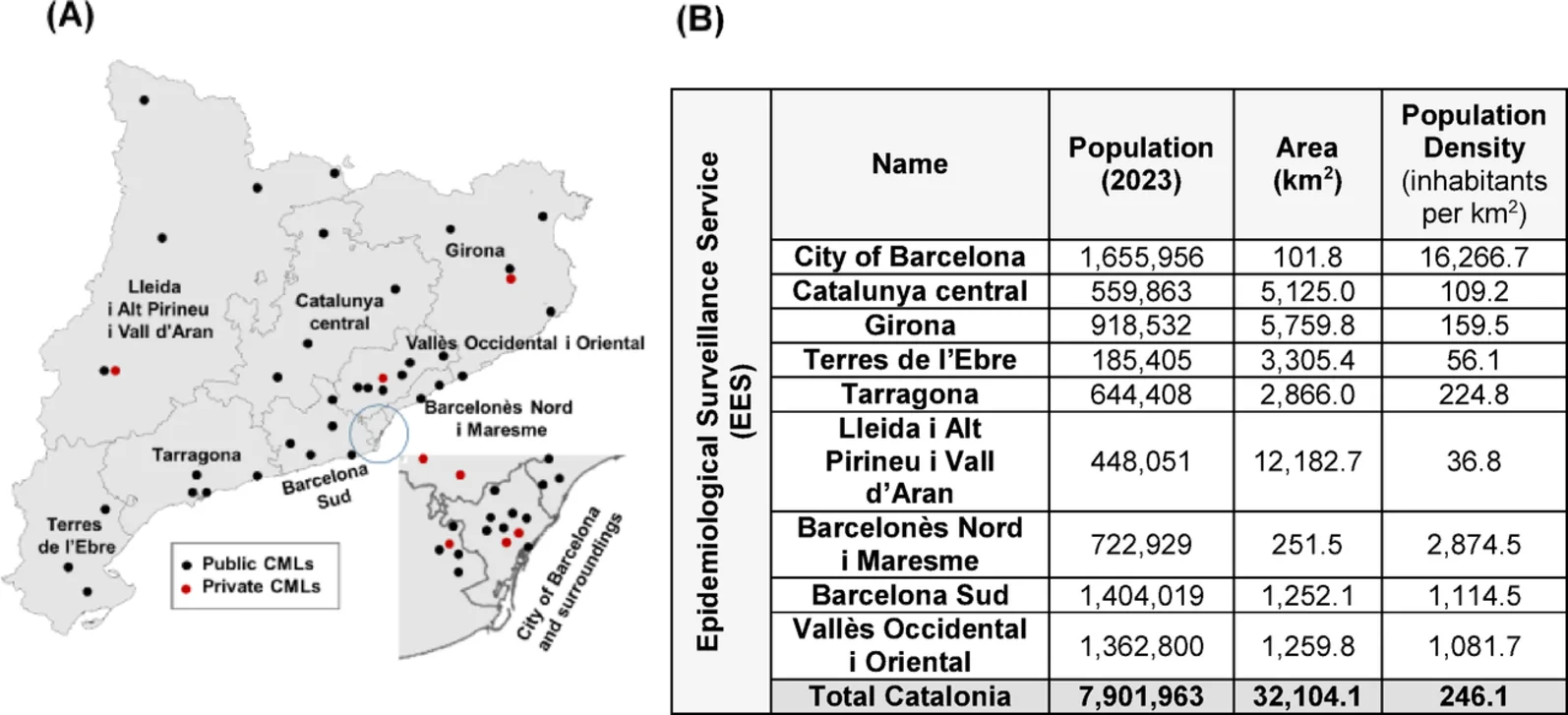
Epidemiological Surveillance Network of Catalonia. (**A)** Map of geographical distribution of epidemiological surveillance services of Catalonia and clinical microbiology laboratories (CMLs). (**B)** Population demographics and geographical extension data of each epidemiological surveillance service. Source: Subdirectorate General for Public Health Surveillance and Emergency Response (SGVRESP) and Statistical Institute of Catalonia (IDESCAT). Source: Subdirectorate General for Public Health Surveillance and Emergency Response (SGVRESP) and StatisticalInstitute of Catalonia (IDESCAT)

Catalonia is a region of Spain with 7,901,963 inhabitants in 2023. The XVEC is organized into nine Epidemiological Surveillance Services (ESS) aligned with the Catalan health structure and defined by historical and territorial considerations (Fig. 1A). Each ESS has its own demographic and territorial characteristics (Fig. 1B).

The MRSC currently includes 53 clinical microbiology laboratories (CMLs), both public and private, serving 56 hospitals. Hospital bed capacity varied, with 28 hospitals (50.0%) having fewer than 200 beds, 20 (35.7%) having 200–499 beds, 6 (10.7%) having 500–999 beds, and 2 (3.6%) having ≥ 1000 beds. Overall, 98.4% of acute care centres included in the Catalan Public Health System (SISCAT) reported to the MRSC, covering 98.3% of total acute care beds [8] .

The geographical distribution of the CMLs is shown in Fig. 1A. CMLs report confirmed infectious disease cases and their AMR data, contribute to AMR trend monitoring, and support both local and regional management strategies.

### Microorganisms under AMR surveillance and data collection

According to current regional legislation and the Catalan AMR surveillance protocol, AMR monitoring is structured into two modalities: **(1)** **Individualised resistance surveillance**, focusing on: *Campylobacter jejuni*, *Mycobacterium tuberculosis* complex, *Neisseria gonorrhoeae*, *Neisseria meningitidis*, *Salmonella* spp., and *Streptococcus pneumoniae*; and** (2)** **Cumulative resistance surveillance** targeting key healthcare-associated microorganisms: *Acinetobacter baumannii*,* Enterococcus faecium*,* Enterococcus faecalis*,* Escherichia coli*,* Klebsiella pneumoniae*,* P. aeruginosa*, methicillin-susceptible *S. aureus* (MSSA), methicillin-resistant *S. aureus* (MRSA), as well as other relevant microorganisms such as *Streptococcus agalactiae*, and *Streptococcus pyogenes*.

Cumulative data are collected annually in aggregate format. CMLs report the number of strains tested and classify them as susceptible, susceptible with increased exposure, or resistant to the antibiotics under surveillance [9]. Additionally, CMLs with the capacity to detect resistance mechanisms (RMs), such as extended-spectrum beta-lactamases (ESBLs), carbapenemases, and glycopeptide-resistant strains — as well as multidrug-resistant (MDR), extensively drug-resistant (XDR), and pandrug-resistant (PDR) strains, according to the definitions proposed by Magiorakos et al. [10], also report these information. Only one isolate per patient and per infectious episode is reported, and only isolates obtained from sterile sites (e.g. blood, cerebrospinal fluid, pleural fluid) are included. Antimicrobial susceptibility is interpreted according to the annual EUCAST breakpoint updates. Moreover, each CML completes a form to assess compliance with the specific criteria for each microorganism and antibiotic under surveillance. Non-compliant AMR data are excluded from the analysis to ensure statistical reliability. The aggregated format and the list of antibiotics and RMs assessed for each microorganism are available as Supplementary material (Supplementary Text S1 and Supplementary Table S1).

To this end, a survey was developed to assess the complexity, characteristics, and laboratory information systems (LIS) of each CML. The survey is provided as Supplementary material (Supplementary Text S2). This will help to understand the local context, enhance the quality of the AMR surveillance network through the implementation of automated and individualised reports, and designate regional reference CMLs responsible for sequencing strains of public health importance.

### Epidemiological SurveillanceIinformation System of Catalonia (SIVEC)

The SIVEC, now under development, will provide real-time integration of clinical, epidemiological, and microbiological data within a single interface for monitoring notifiable infectious diseases and AMR.

The system’s main functionalities include the management of notifications, cases and laboratory test results related to notifiable diseases. The SIVEC incorporates ICD-10 (International Statistical Classification of Diseases and Related Health Problems, 10th Revision), SNOMED CT (Systematized Nomenclature of Medicine – Clinical Terms) and LOINC (Logical Observation Identifiers Names and Codes) standards, and is interconnected with primary care services, hospitals and laboratories through an integrated health information platform, enabling the linkage of clinical and microbiological data.

To support and strengthen antimicrobial resistance (AMR) surveillance, a catalogue of microorganisms, specimens, locations, techniques, antibiotics and results was developed using SNOMED CT, together with a serology catalogue based on LOINC.

The use of standardised terminologies will enhance the quality, consistency and interoperability of health information systems. It will facilitate data exchange between institutions, support surveillance and data analysis, and enable the integration of multiple data sources. In addition, it will improve the comparability of health data across different settings.

### Data analysis and dissemination of outputs

The analysis focuses on the number of bacterial strains tested annually, together with their susceptibility interpretations, RMs, and resistance patterns (including multidrug resistance [MDR], extensively drug-resistant [XDR], and pan-drug resistance [PDR]). Each year, the data are thoroughly analysed and discussed with CMLs before publication, in reports that present both current and retrospective AMR data. All reports are available on the institutional website (https://salutpublica.gencat.cat/ca/inici/). The data are also used to inform the development of healthcare practices and antibiotic prescribing policies, led by the Programme for the Optimisation of Antimicrobials in the Health System of Catalonia (PROACat) and Spain’s National Plan against Antibiotic Resistance (PRAN). These data support the analysis of antibiotic consumption, prescribing practices and the implementation of public awareness campaigns aimed at promoting appropriate antibiotic use. In addition, they are used as academic support for training programmes aimed at accrediting clinicians in the rational use and optimisation of antibiotics [11, 12].

### Statistical analysis

Temporal trends in antimicrobial resistance were assessed for each microorganism over the study period (2016–2023). For each year, resistance proportions were calculated as the number of resistant antimicrobial susceptibility test results divided by the total number of tests performed.

Trends over time were evaluated using binomial logistic regression models with calendar year included as a continuous variable. Results are presented as odds ratios (ORs) per year, representing the annual change in the odds of resistance, together with corresponding 95% confidence intervals (95% CI) and *p*-values. An OR > 1 indicates an increasing trend, whereas an OR < 1 indicates a decreasing trend. A *p*-value < 0.05 was considered statistically significant.

All statistical analyses were performed using IBM SPSS Statistics (version 24.0, IBM Corp., Armonk, NY, USA), and figures were generated using Microsoft Excel.

## Results

### Present of AMR data in catalonia

**Fig. 2:**
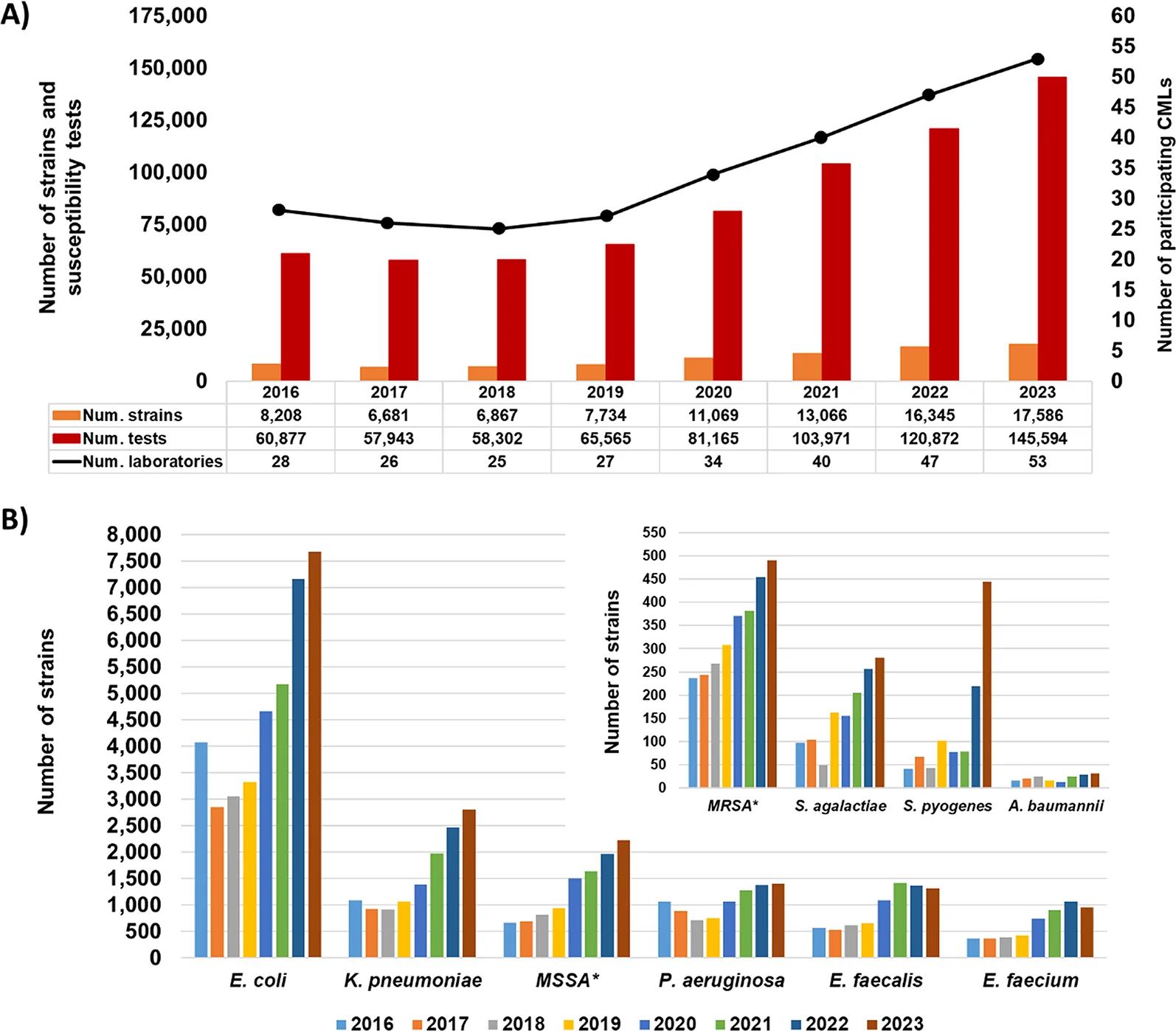
Evolution of microbiological strains, susceptibility tests, participating laboratories, and microorganisms notified, Catalonia (Spain), 2016-2023.(A) Annual evolution of the number of microbiological strains, susceptibility tests, and participating laboratories, 2016–2023. (Black line) = number of clinical microbiology laboratories per year; (orange columns) = number of notified microbiological strains; (red columns) = number of antimicrobial susceptibility tests performed. (B) Evolution of notified microorganisms, 2016–2023. Surveillance covered *Escherichia coli*, *Klebsiella pneumoniae*, MSSA, *Pseudomonas aeruginosa*, *Enterococcus faecalis*, *Enterococcus faecium*, MRSA, *Streptococcus agalactiae*, *Streptococcus pyogenes*, and *Acinetobacter baumannii*. CMLs: clinical microbiology laboratories; MSSA: methicillin-susceptible *Staphylococcus aureus*; MRSA: methicillin-resistant *Staphylococcus aureus*

Adherence by CMLs to AMR reporting to the MRSC has been gradual. In 2016, the first year of mandatory notification, 28 centres participated in AMR surveillance. The number of participating laboratories increased steadily, reaching 53 in 2023. This 89.3% increase directly influenced the volume of data collected, with a total of 87,556 invasive microbiological strains and their susceptibility profiles (694,289 tests) reported to the AMR database between 2016 and 2023 (Fig. 2A and Supplementary Table S2). Among the microorganisms under surveillance, *E. coli* accounted for the largest number of notifications (37,999 invasive strains), followed by *K. pneumoniae* (12,626), MSSA (10,464) and *P. aeruginosa* (8553). Fewer notifications were reported for *E. faecalis* (7561), *E. faecium* (5224), MRSA (2749), *S. agalactiae* (1308), *S. pyogenes* (1072), and *A. baumannii* (174) (Fig. 2B and Supplementary Table S2).

**Fig. 3:**
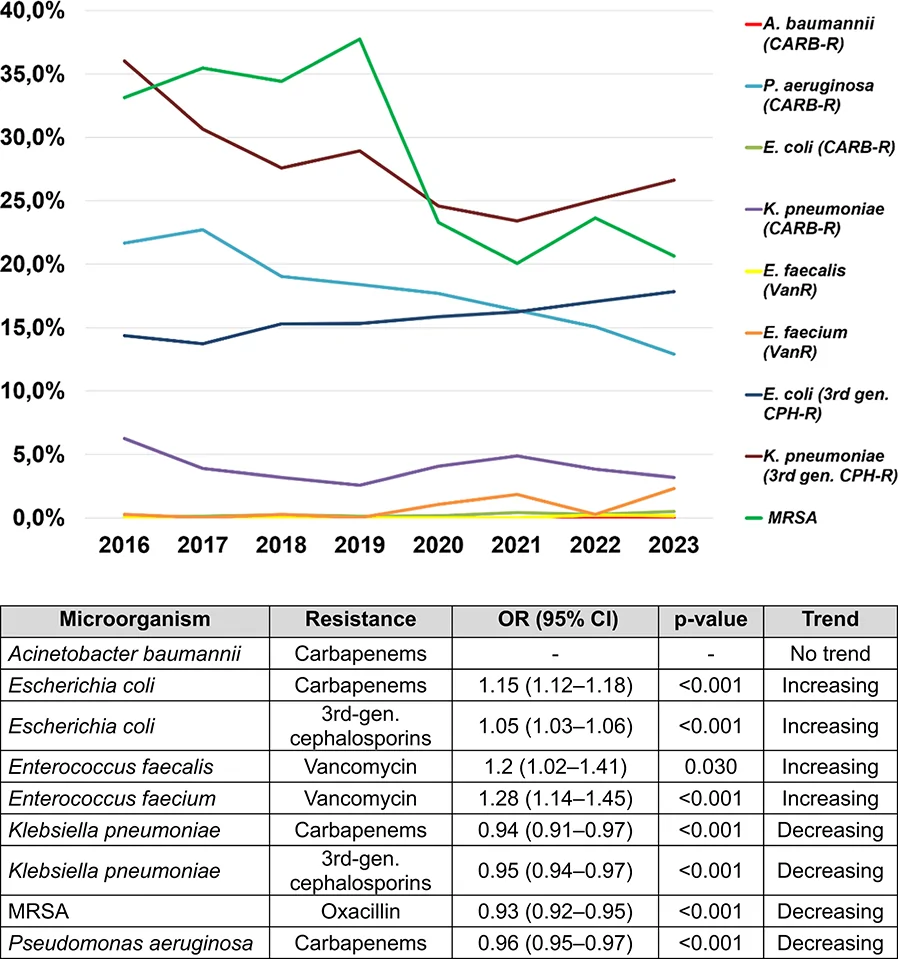
Temporal evolution of resistance proportions based on antimicrobial susceptibility testing by microorganism, Catalonia (Spain), 2016–2023. Temporal trends were assessed using binary logistic regression models, with calendar year included as a continuous variable. Analysis were perfomed using IBM SPSS Statistics (version 24.0, IBM Corp., Armonk, NY, USA). Odds ratios (ORs) represent the annual change in the odds of resistance, and p-values indicate statistical significance. An OR > 1 indicates an increasing trend, whereas an OR < 1 indicates a decreasing trend; p-values < 0.05 were considered statistically significant. Models were not estimable for *A. baumannii* due to the absence of resistant cases. Figures were created using Microsoft Excel

To assess temporal changes in antimicrobial resistance, binary logistic regression models were applied for key microorganisms over the study period (2016–2023), and resistance proportions were examined over time (Fig. 3; Supplementary Table S3).

Carbapenem resistance decreased in *P. aeruginosa* (21.7% in 2016 to 12.9% in 2023; OR 0.96, 95% CI 0.95–0.97; *p* < 0.001) and *K. pneumoniae* (6.3 to 3.2%; OR 0.94, 95% CI 0.91–0.97; *p* = 0.001), whereas *E. coli* showed a low but increasing trend (0.07 to 0.52%; OR 1.15, 95% CI 1.12–1.18; *p* < 0.001).

Vancomycin resistance increased in *E. faecium* (0.27 to 2.30%; OR 1.28, 95% CI 1.14–1.45; *p* < 0.001) and remained low in *E. faecalis*, with only sporadic cases detected at the end of the study period (OR 1.20, 95% CI 1.02–1.41; *p* = 0.030).

Third-generation cephalosporin resistance increased in *E. coli* (14.4 to 17.8%; OR 1.05, 95% CI 1.03–1.06; *p* < 0.001), whereas *K. pneumoniae* showed an overall decrease despite some fluctuations (36.0 to 26.6%; OR 0.95, 95% CI 0.94–0.97; *p* < 0.001). MRSA proportions also declined over time, from 33.1 to 20.6% (OR 0.93, 95% CI 0.92–0.95; *p* < 0.001).

Carbapenem resistance in *A. baumannii* remained negligible, with no resistant isolates detected during the study period.

Temporal trends were assessed using binary logistic regression models, with calendar year included as a continuous variable. Analysis were perfomed using IBM SPSS Statistics (version 24.0, IBM Corp., Armonk, NY, USA). Odds ratios (ORs) represent the annual change in the odds of resistance, and p-values indicate statistical significance. An OR > 1 indicates an increasing trend, whereas an OR < 1 indicates a decreasing trend; *p*-values < 0.05 were considered statistically significant. Models were not estimable for *A. baumannii* due to the absence of resistant cases. Figures were created using Microsoft Excel.

Abbreviations: 3rd gen. CPH-R; third generation cephalosporins resistance;CARB-R; carbapenem resistance; MRSA, methicillin-resistant *S. aureus;* VanR, vancomycin resistance; Carbapenems included meropenem, ertapenemand imipenem; 3rd generation cephalosporin includes cefotaxime; and glycopeptides refer to vancomycin resistance in *Enterococcus* spp.

### Survey analysis

To assess the characteristics, complexity, and laboratory information systems (LIS) of each CMLs, a specific survey with different sections was developed (Supplementary Text S2).

The survey focused on four key areas: (1) Microbiological reporting (EUCAST use and the distinction between infection and colonization); (2) resistance mechanism (RM) analysis (performed locally or externally); (3) laboratory complexity based on AMR testing techniques; and (4) laboratory information systems (LIS).

The main results are shown in Table 1. All CMLs completed the survey and all (100%; 53/53) used EUCAST guidelines for determining antibiotic susceptibility, although only 56.6% used the current 2023 guideline. In addition, 94.3% (50/53) of these CMLs ensured that reported strains were obtained from sterile sites and attributable to a single patient/infectious event.

Regarding laboratory complexity, three categories were established considering the catalogue of microbiological techniques (phenotypical and genotypical) of each CML: Level 1 AMR laboratories (L1) perform only phenotypic techniques (e.g. disk diffusion susceptibility testing, manual and automated broth dilution methods, concentration gradient diffusion assays, chromogenic media, and immunochromatographic tests); Level 2 AMR laboratories (L2) perform some or all L1 techniques and have also implemented molecular methods (e.g. PCR) and mass spectrometry (MALDI–TOF); and Level 3 AMR laboratories (L3) perform L1 and L2 methods and, in addition, can undertake advanced genomic analyses such as Sanger sequencing, multilocus sequence typing (MLST), and/or whole genome sequencing (WGS).

Based on these definitions, Catalonia has 10 L1 CMLs (18.9%), 35 L2 CMLs (66.0%), and 8 L3 CMLs (15.1%). The majority of invasive microbiological notifications were reported by L2 (57.4%) and L3 laboratories (39.4%), whereas L1 laboratories contributed a smaller proportion (3.2%) (Table 1).

**Table 1:** Antimicrobial resistance surveillance criteria, data aspects, laboratory techniques, and level of clinical microbiological laboratories, Catalonia (Spain), 2023 (*n* = 53 CMLs)

Survey results	Yes		No		NA		
	*N*	(%)	*N*	(%)	*N*		(%)
EUCAST guidelines	53	(100.0)	0	(0.0)	0	(0.0)	
EUCAST guidelines (2023)	30	(56.6)	23	(43.4)	0	(0.0)	
Single patient/infection	50	(94.3)	1	(1.9)	2	(3.8)	
Sterile samples	50	(94.3)	1	(1.9)	2	(3.8)	
Own centre	53	(100.0)	0	(0.0)	0	(0.0)	
Only infections / no colonisations	51	(96.2)	1	(1.9)	1	(1.9)	
Total year (2023)	52	(98.1)	0	(0.0)	1	(1.9)	
Multidrug resistance (MDR/XDR/PDR)	39	(73.6)	13	(24.5)	1	(1.9)	

Resistance mechanisms	Yes	(%)	No	(%)	NA	(%)	
Resistance mechanisms (own centre)	49	(92.5)	4	(7.5)	0	(0.0)	
Resistance mechanisms (other centre)	0	(0.0)	0	(0.0)	0	(0.0)	
Multidrug resistance (MDR/XDR/PDR)	39	(73.6)	13	(24.5)	1	(1.9)	

Laboratory techniques	Disponibility		No disponibility		CML Level N (%)		% TN
	N	(%)	N	(%)			
Disk diffusion susceptibility test	53	(100.0)	0	(0.0)	L1 *N* = 10 (18.9)		3.2
Broth dilution method (manual)	36	(67.9)	17	(32.1)			
Broth dilution method (automatized)	49	(92.5)	4	(7.5)			
Concentration gradient diffusion assay	42	(79.2)	11	(20.8)			
Chromogenic media	48	(90.6)	5	(9.4)			
Immunochromatographic test	46	(86.8)	7	(13.2)			
Polymerase Chain Reaction (PCR)	43	(81.1)	10	(18.9)	L2 *N* = 35 (66.0)		57.4
Multiplex PCR	41	(95.3)	NA				
End-point PCR	2	(4.7)	NA				
Mass spectrometry (MALDI-TOF)	42	(79.2)	11	(20.8)			
First-generation sequencing (Sanger)	8	(15.1)	45	(84.9)	L3 *N* = 8 (15.1)		39.4
Multilocus sequence typing (MLST)	8	(15.1)	45	(84.9)			
Whole genome sequencing (WGS)	8	(15.1)	45	(84.9)			

Abbreviations: EUCAST, European Committee on Antimicrobial Susceptibility Testing; L1, level 1; L2, level 2; L3, level 3; CML, Clinical microbiology laboratory; MDR, Multiple-drug resistance; N, number; NA, not answered; PDR, pan-drug resistance; and XDR, extensively drug-resistance; %TN, proportion of invasive microbiological notifications according to CML level classification.

Forty-nine CMLs (92.5%) were able to detect RMs, and 39 (73.6%) monitored multidrug-resistant strains. In Enterobacterales (*E. coli* and *K. pneumoniae*), the most frequently analysed RMs were OXA-48, VIM, NDM and IMP (49/49; 100%) and KPC (48/49; 98.0%). For non-fermenter microorganisms, VIM was assessed in 47 CMLs (95.9%) for *P. aeruginosa* and in 39 (79.6%) for *A. baumannii*. NDM was evaluated in 39 CMLs (79.6%) for *A. baumannii* isolates, while IMP was tested in 39 (79.6%) and 42 (85.7%) CMLs for *A. baumannii* and *P. aeruginosa*, respectively. KPC in *P. aeruginosa* was included in the routine of 44 CMLs (89.8%).

Among Gram-positive microorganisms, VanA and VanB phenotypes in *E. faecalis* and *E. faecium* isolates were assessed by 28 CMLs (57.1%). M, iMLSB and cMLSB phenotypes in *S. agalactiae* and *S. pyogenes* were evaluated in 25 CMLs (51.0%) (Table 2**)**.

**Table 2:** Number of clinical microbiology laboratories with capacity to detect carbapenemase and other antimicrobial resistance mechanisms, Catalonia (Spain), 2023 (*n* = 49 CMLs)

Resistance mechanism		Microorganism							
		Enterobacterales				Non-fermenters			
Carbapenemases	Ambler class	*E. coli*		*K. pneumoniae*		*A. baumannii*		*P*. *aeruginosa*	
		N	(%)	N	(%)	N	(%)	N	(%)
OXA-40	D	NA		NA		7	(14.3)	11	(22.4)
OXA-48 like	D	49	(100.0)	49	(100.0)	NA		NA	
OXA-58	D	NA		NA		6	(12.2)	NA	
OXA-23	D	NA		NA		7	(14.3)	NA	
OXA-198	D	NA		NA		NA		9	(18.4)
VIM	B	49	(100.0)	49	(100.0)	39	(79.6)	47	(95.9)
NDM	B	49	(100.0)	49	(100.0)	39	(79.6)	39	(79.6)
IMP	B	49	(100.0)	49	(100.0)	39	(79.6)	42	(85.7)
SPM	B	NA		NA		NA		13	(26.6)
GIM	B	NA		NA		NA		18	(36.7)
KPC	A	48	(98.0)	48	(98.0)	NA		44	(89.8)
GES	A	19	(38.8)	19	(38.8)	NA		18	(36.7)

Glycopeptides RMs		*E. faecalis*		*E. faecium*	
		N	(%)	N	(%)
vanA phenotype		28	(57.1)	28	(57.1)
vanB phenotype		28	(57.1)	28	(57.1)

Macrolide and MLS RMs	*S.agalactiae*		*S. pyogenes*	
	N	(%)	N	(%)
M phenotype	25	(51.0)	25	(51.0)
iMLSB phenotype	25	(51.0)	25	(51.0)
cMLSB phenotype	25	(51.0)	25	(51.0)

N, number CMLs; NA, not applicable (not described or extremely rare); MLSB, Macrolide, Lincosamide, and Streptogramin B; RMs, Resistant mechanisms (%), percentage.

### Laboratory information system (LIS)

All CMLs reported having a laboratory information system (LIS). In total, 53 CMLs used 13 different LIS platforms, designated with the letters A–M. The most common was LIS A, used by 20 CMLs (37.7%), followed by LIS B (8 CMLs; 15.1%) and LIS C (6 CMLs; 11.3%). The remaining systems were distributed as follows: LIS D (5 CMLs; 9.4%), LIS E (3; 5.7%), LIS F, G and H (2 each; 3.8%), and LIS I, J, K, L and M (1 each; 1.9%).

## Discussion

Across Europe, numerous initiatives have been implemented to strengthen AMR surveillance from an integrated and real-time approaches [13–15]. A notable example is the Dutch national surveillance system, the Infectious Diseases Surveillance Information System for Antimicrobial Resistance (ISIS-AR). ISIS-AR monitors AMR trends and outbreaks, detects novel resistance mechanisms, and supports public health decisions, empirical treatment guidelines, and research to improve quality and reduce healthcare costs through a universal and standardized coding nomenclature. However, in contrast to Catalonia, the enrolment in the ISIS-AR remains voluntary which may limit national representativeness. Conversely, Greece is currently undergoing an extensive process of harmonization and standardization of AMR surveillance. This is driven by several factors, including limitations in laboratory diagnostics capabilities, the adoption of EUCAST standards and clinical breakpoints, and the implementation of LIS across CMLs. At national level, Spain has made notable advances, particularly through the Microbiological Surveillance Network in the Valencian Community (RedMIVA), which set a significant precedent [16]. The RedMIVA network was pioneering, establishing a centralized system that integrated microbiological test results, including AMR data, from hospital laboratories across its territory, into epidemiological surveillance data for the transmission of case information related to notifiable diseases. To standardize data, master tables were created with numeric code, short descriptive code, and long descriptive definitions. Likewise, epidemiological surveillance in Catalonia, including AMR monitoring, is expected to become fully automated in the coming months. The SIVEC platform will integrate clinical, epidemiological, and laboratory data into a single interface. Unlike RedMIVA, laboratory information will be standardized using SNOMED CT for medical and microbiological terminology—including AMR-related terms—and LOINC for laboratory observations and serological interpretations, enhancing both interoperability and universality. The use of standardized coding systems will also facilitates the inclusion of emerging or under-monitored microorganisms, such as *Proteus* spp., *Candida* spp. and *Candidozyma auris* [17]. These advances will allow individualised epidemiological surveillance of all microorganisms, supporting comprehensive AMR monitoring that incorporates the full clinical and epidemiological context of each case. Furthermore, we consider it essential to implement targeted surveillance of these microorganisms, as their incidence is high and comparable to—or even exceeds—that of several notifiable diseases, which may have significant public health implications. In Catalonia, the incidence of invasive infections due to *E. coli* (96.9 per 100,000) and *K. pneumoniae* (35.4) are comparable to—or even exceed—respiratory syncytial virus (RSV) (69.7), *Campylobacter* spp. (84.8), and *Salmonella* spp. (26.8) [8]. In addition, both *E. coli* and *K. pneumoniae* continue to exhibit high resistance levels, reinforcing the need for targeted surveillance of these pathogens [18–20]. The possibility of visualizing, through graphs, all aspects collected in AMR surveillance is also planned, taking as an example the Catalan sentinel surveillance program for community-acquired respiratory infections (SIVIC) (https://sivic.salut.gencat.cat/ira_ap_micro).

The results highlight a high level of coverage and representativeness of AMR surveillance in Catalonia, with participation from most acute care centres and a progressive increase in contributing laboratories over time, leading to a substantial growth in available data and reinforcing the robustness of the system.

Regarding AMR trends, the observed patterns indicate changes in resistance over time. The decrease in carbapenem resistance in *P. aeruginosa* and *K. pneumoniae*, together with the reduction in MRSA proportions, may reflect the impact of infection control measures and antimicrobial stewardship programmes implemented during the study period within the Catalan PROA framework. In contrast, increasing trends in *E. coli* suggest evolving resistance patterns and highlight the need for further investigation into their epidemiological drivers, including sources and transmission dynamics.

Vancomycin resistance in *E. faecium* increased, although from low baseline levels, supporting the need for continued monitoring, while *E. faecalis* remained largely stable. These findings underline the importance of comprehensive and standardised surveillance systems, such as SIVEC, to better characterise evolving resistance patterns and inform timely public health responses.

The classification of CMLs by level of complexity may support the development of structured laboratory networks, in which L1 laboratories are connected to L2 laboratories and these, in turn, to L3 regional reference laboratories. This model would facilitate disease control, improve coordination across the network and support the transition towards genomic AMR surveillance, in line with national and European frameworks, including RedLabRA, the Spanish Antibiogram Committee (COESANT), and ECDC recommendations.

Some limitations should be considered. Resistance mechanisms not routinely tested across all laboratories may be underreported, although this mainly affects a small number of low-activity centres (*n* = 4, 0.3%). In addition, heterogeneity in LIS and the lack of standardised recording of certain data, such as macrolide and MLS resistance phenotypes, limit their use for retrospective analysis. This variability reflects the diversity of LIS across CMLs, including differences in providers, structure and data capture practices, which remains a key challenge for data standardisation and integration. Another limitation is the lack of systematic surveillance of certain resistance mechanisms, such as ESBLs, as monitoring has mainly prioritised carbapenemase-producing organisms, potentially limiting the assessment of the overall resistance burden.

These limitations are expected to be addressed through the implementation of SIVEC, which will improve data standardisation, integration and completeness, and strengthen the capacity to detect and respond to emerging AMR threats in a timely manner.

## Conclusions

This study provides a comprehensive overview of AMR surveillance coverage in Catalonia, integrating public and private CMLs and offering representative data on resistance patterns and diagnostic capacity. It represents the first regional assessment combining laboratory practices, diagnostic capabilities and surveillance reporting across a heterogeneous network, establishing a baseline for the transition to automated, real-time surveillance through SIVEC.

All laboratories follow EUCAST standards, although further efforts are needed to ensure the uniform and timely implementation of annual updates across all sites. Regarding the phenotypic detection of resistance mechanisms, additional work is required to ensure that all laboratories are able to perform these analyses. The proposed classification of laboratories by complexity, together with the designation of regional reference and support laboratories, would strengthen coordination and diagnostic capacity.

The implementation of the SIVEC platform represents a critical step towards automated, integrated, and real-time AMR monitoring. Its capacity to incorporate clinical, epidemiological, and microbiological data will facilitate early detection of emerging resistance mechanisms, improve interoperability, and support evidence-based public health decision-making.

## Supplementary Information

Below is the link to the electronic supplementary material.

Supplementary Material 1

Supplementary Material 2

Supplementary Material 3

Supplementary Material 4

Supplementary Material 5

## Data Availability

No datasets were generated or analysed during the current study.

## Abbreviations

AMR: Antimicrobial resistance
ASPCAT: Public Health Agency of Catalonia
CML: Clinical Microbiology Laboratory
COESANT: Spanish Antibiogram Committee
ECDC: European Centre for Disease Prevention and Control
ESBL: Extended Spectrum Beta-Lactamases
ESS: Epidemiological surveillance services
EUCAST: European Committee on Antimicrobial Susceptibility Testing
IDESCAT: Statistical Institute of Catalonia
LIS: Laboratory information system
LOINC: Logical Observation Identifiers Names and Codes
MALDI-TOF: Matrix-Assisted Laser Desorption Ionization Tome of Light
MDR: Multidrug resistance
MLST: Multi locus sequence typing
MSSA: Methicillin-susceptible Staphylococcus aureus
MRSA: Methicillin-resistant Staphylococcus aureus
MRSC: Microbiological Reporting System of Catalonia
PDR: Pan-drug resistance
PRAN: National Plan Against Antibiotic Resistance
PROACat: Programme for the Optimisation of Antimicrobials of Catalonia
RedLabRA: National AMR Surveillance Programme
RedMIVA: Microbiological Surveillance Network in the Valencian Community
RM: Resistance mechanism
SDR: Statutory Disease Reports
SISCAT: Catalan Public Health System
SGVRESP: Subdirectorate General for Public Health Surveillance and Emergency Response Health
SIVEC: Epidemiological Surveillance Information System of Catalonia
SNOMED: Systematized Nomenclature of Medicine
WHO: World Health Organization
XDR: Extensively drug-resistant
XVEC: Epidemiological Surveillance Network of Catalonia
