# Supplementary material for: The present and the future of antimicrobial resistance surveillance in Catalonia (Spain), 2024: from manual to automatic notification
Source: Antimicrob Resist Infect Control. 2026 Jun 30;15:125. doi: 10.1186/s13756-026-01781-0 (PMC13644157; doi:10.1186/s13756-026-01781-0)
Supplement: Supplementary file 1 [file 13756_2026_1781_MOESM1_ESM.docx]

|  | **Number of tested strains (Number of susceptibility tests)** | | | | | | | | |  |
| --- | --- | --- | --- | --- | --- | --- | --- | --- | --- | --- |
|  | **2016** | **2017** | **2018** | **2019** | **2020** | **2021** | **2022** | **2023** | **TOTAL** |  |
| ***Acinetobacter baumannii*** | 16 (66) | 20 (83) | 25 (115) | 16 (68) | 13 (57) | 24 (105) | 29 (138) | 31 (149) | 174 (781) |  |
| ***Enterococcus faecalis*** | 572 (1,839) | 534 (1,970) | 622 (2,042) | 648 (2,250) | 1,085 (3,810) | 1,410 (4,473) | 1,371 (4,526) | 1,319 (4,616) | 7,561 (25,526) |  |
| ***Enterococcus faecium*** | 366 (1,265) | 364 (1,276) | 386 (1,253) | 423 (1,514) | 747 (2,466) | 908 (2,891) | 1,071 (3,571) | 959 (3,389) | 5,224 (17,624) |  |
| ***Escherichia***  ***coli*** | 4,079 (26,486) | 2,858 (24,610) | 3,052 (26,918) | 3,330 (29,318) | 4,664 (32,432) | 5,180 (45,491) | 7,166 (56,790) | 7,670 (66,190) | 37,999 (308,235) |  |
| ***Klebsiella pneumoniae*** | 1,086 (8,574) | 928 (8,207) | 914 (7,248) | 1,065 (8,389) | 1,392 (10,462) | 1,980 (13,096) | 2,463 (18,494) | 2798 (21,563) | 12,626 (96,033) |  |
| ***Pseudomonas aeruginosa*** | 1,064 (11,060) | 893 (9,100) | 715 (7,238) | 751 (7,840) | 1,071 (9,782) | 1,283 (11,950) | 1,376 (11,397) | 1,400 (13,201) | 8,553 (81,568) |  |
| **MSSA***** | 666 (8,182) | 690 (8,989) | 818 (9,944) | 947 (11,534) | 1,508 (17,158) | 1,640 (20,762) | 1,969 (19,905) | 2,226 (28,142) | 10,464 (124,616) |  |
| **MRSA*** | 236 (2,979) | 243 (3,173) | 268 (3,269) | 307 (3,835) | 370 (4,292) | 381 (4,344) | 454 (4,621) | 490 (6,155) | 2,749 (32,668) |  |
| ***Streptococcus agalactiae*** | 97 (302) | 104 (304) | 49 (147) | 162 (513) | 155 (474) | 205 (620) | 256 (768) | 280 (838) | 1,308 (3,996) |  |
| ***Streptococcus pyogenes*** | 42 (124) | 67 (201) | 43 (129) | 101 (304) | 77 (232) | 79 (239) | 219 (662) | 444 (1,351) | 1,072 (3,242) |  |
| **Number**  **labs/centers** | **28** | **26** | **25** | **27** | **34** | **40** | **47** | **53** | **53** |  |
| **TOTAL** | **8,208 (60,877)** | **6,681 (57,943)** | **6,867 (58,302)** | **7,734 (65,565)** | **11,069 (81,165)** | **13,066 (103,971)** | **16,345 (120,872)** | **17,586 (145,594)** | **87,556 (694,289)** |  |
| ***** MSSA*,*** **Methicillin-susceptible *Staphylococcus aureus;* MRSA*,* Methicillin-resistant *Staphylococcus aureus.*** | | | | | | | | | | |

**Supplementary table 1.** Number of tested strains and susceptibility tests (2016-2023) Catalonia (Spain).
