# Supplementary material for: The present and the future of antimicrobial resistance surveillance in Catalonia (Spain), 2024: from manual to automatic notification
Source: Antimicrob Resist Infect Control. 2026 Jun 30;15:125. doi: 10.1186/s13756-026-01781-0 (PMC13644157; doi:10.1186/s13756-026-01781-0)
Supplement: Supplementary file 2 [file 13756_2026_1781_MOESM2_ESM.docx]

**Supplementary Text S1.** Aggregated antibiotic susceptibility data (Year 2023)

(Fill in fields)

- Health centre / Centre code
- Responsible facultative for the declaration
- Email
- Contact phone (extension)

1. **Regarding the breakpoints used to classify strains as susceptible, susceptible with increased exposure, or resistant. In 2023, did you follow the breakpoints according to the criteria described in this year’s updated EUCAST guidelines?**

- YES
- NO (specify the guideline used) __________________________

2. **Regarding the notified microorganisms. Could you indicate the year of the guideline used?**

| **Microorganism** | **EUCAST Guidelines (Yes/No*)** | **EUCAST (year used)** | ***Specify guideline if different from EUCAST** |
| --- | --- | --- | --- |
| *Acinetobacter baumannii* |  |  |  |
| *Enterococcus faecalis* |  |  |  |
| *Enterococcus faecium* |  |  |  |
| *Escherichia coli* |  |  |  |
| *Klebsiella pneumoniae* |  |  |  |
| *Pseudomonas aeruginosa* |  |  |  |
| *Staphylococcus aureus* (MSSA) |  |  |  |
| *Methicillin-resistant Staphylococcus aureus* (MRSA) |  |  |  |
| *Streptococcus agalactiae* |  |  |  |
| *Streptococcus pyogenes* |  |  |  |

**Observations:**

3. **Do all the strains you report correspond to a single patient and infection process?**

- YES
- NO*

**Observations:**

**In case the reported strains do not correspond to the same patient and infection process, you must inform in the form the number of non-duplicate strains with their antibiotic sensitivity profile for each microorganism.*

4. **Do the analyzed samples correspond only to sterile samples for all reported microorganisms?**

- YES
- NO

**Observations:**

5. **Are all the strains you report isolated in your center?**

- YES
- NO*

**Observations:**

**If not, you must indicate in the form how many strains were not isolated in your center and which center they came from.*

6. **Do all the strains you report correspond to...**

- Only infection cases
- Colonizations are also included*

**Observations:**

**In principle, colonizations are not considered for the declaration. However, if you also report colonizations, you can indicate in the form the percentage that corresponds to infection and colonization.*

7. **Do all strains for which antibiotic sensitivity is declared correspond to the total strains of the reported year in the form?**

- YES
- NO*

***Observations:**

8. **Do you carry out resistance mechanism studies in your own center?**

- YES*
- NO

*If yes, which microorganisms?

(Mark the microorganism and indicate which resistance mechanisms (RM) are studied)

- ***Acinetobacter baumannii***

|  | **Yes** | **No** |
| --- | --- | --- |
| Carbapenemases (OXA-40) |  |  |
| Carbapenemases (OXA-58) |  |  |
| Carbapenemases (OXA-23) |  |  |
| Metallo-β-lactamases (VIM) |  |  |
| Metallo-β-lactamases (NDM) |  |  |
| Metallo-β-lactamases (IMP) |  |  |
| Others (specify) |  |  |

- ***Enterococcus faecalis***

|  | **Yes** | **No** |
| --- | --- | --- |
| vanA phenotype |  |  |
| vanB phenotype |  |  |
| Others (specify) |  |  |

- ***Enterococcus faecium***

|  | **Yes** | **No** |
| --- | --- | --- |
| vanA phenotype |  |  |
| vanB phenotype |  |  |
| Others (specify) |  |  |

- ***Escherichia coli***

|  | **Yes** | **No** |
| --- | --- | --- |
| Carbapenemases (OXA-48) |  |  |
| Metallo-β-lactamases (VIM) |  |  |
| Metallo-β-lactamases (NDM) |  |  |
| Metallo-β-lactamases (IMP) |  |  |
| Serine carbapenemases (KPC) |  |  |
| Serine carbapenemases (GES) |  |  |
| Others (specify) |  |  |

- ***Klebsiella pneumoniae***

|  | **Yes** | **No** |
| --- | --- | --- |
| Carbapenemases (OXA-48) |  |  |
| Metallo-β-lactamases (VIM) |  |  |
| Metallo-β-lactamases (NDM) |  |  |
| Metallo-β-lactamases (IMP) |  |  |
| Serine carbapenemases (KPC) |  |  |
| Serine carbapenemases (GES) |  |  |
| Others (specify) |  |  |

- ***Pseudomonas aeruginosa***

|  | **Yes** | **No** |
| --- | --- | --- |
| Carbapenemases (OXA-48) |  |  |
| Metallo-β-lactamases (VIM) |  |  |
| Metallo-β-lactamases (IMP) |  |  |
| Metallo-β-lactamases (SPM) |  |  |
| Metallo-β-lactamases (GIM) |  |  |
| Serine carbapenemases (KPC) |  |  |
| Serine carbapenemases (GES) |  |  |
| Carbapenemases (OXA-40) |  |  |
| Carbapenemases (OXA-198) |  |  |
| Others (specify) |  |  |

- ***Staphylococcus aureus***

|  | **Yes** | **No** |
| --- | --- | --- |
| *mecC* gene detection |  |  |
| Others (specify) |  |  |

- ***Streptococcus agalactiae***

|  | **Yes** | **No** |
| --- | --- | --- |
| M phenotype |  |  |
| iMLSB phenotypes |  |  |
| cMLSB phenotypes |  |  |
| Others (specify) |  |  |

- ***Streptococcus pyogenes***

|  | **Yes** | **No** |
| --- | --- | --- |
| M phenotype |  |  |
| iMLSB phenotypes |  |  |
| cMLSB phenotypes |  |  |
| Others (specify) |  |  |

**Do you select cases for this study (RMs) (severe patient, clinician request, etc.) or do you do it in all cases?**

**Answer:**

9. **Do you send samples to another center to perform resistance mechanism studies?**

- YES*
- NO

*If yes, which microorganisms and which resistance mechanisms?

(Mark the microorganism and indicate which resistance mechanisms are studied)

- ***Acinetobacter baumannii***

| **RMs** | **Yes** | **No** |
| --- | --- | --- |
| Carbapenemases (OXA-40) |  |  |
| Carbapenemases (OXA-58) |  |  |
| Carbapenemases (OXA-23) |  |  |
| Metallo-β-lactamases (VIM) |  |  |
| Metallo-β-lactamases (NDM) |  |  |
| Metallo-β-lactamases (IMP) |  |  |
| Others (specify) |  | |

- ***Enterococcus faecalis***

| **RMs** | **Yes** | **No** |
| --- | --- | --- |
| vanA phenotype |  |  |
| vanB phenotype |  |  |
| Others (specify) |  | |

- ***Enterococcus faecium***

| **RMs** | **Yes** | **No** |
| --- | --- | --- |
| vanA phenotype |  |  |
| vanB phenotype |  |  |
| Others (specify) |  | |

- ***Escherichia coli***

| **RMs** | **Yes** | **No** |
| --- | --- | --- |
| Carbapenemases (OXA-48) |  |  |
| Metallo-β-lactamases (VIM) |  |  |
| Metallo-β-lactamases (NDM) |  |  |
| Metallo-β-lactamases (IMP) |  |  |
| Serine-carbapenemases (KPC) |  |  |
| Serine-carbapenemases (GES) |  |  |
| Others (specify) |  | |

- ***Klebsiella pneumoniae***

| **RMs** | **Yes** | **No** |
| --- | --- | --- |
| Carbapenemases (OXA-48) |  |  |
| Metallo-β-lactamases (VIM) |  |  |
| Metallo-β-lactamases (NDM) |  |  |
| Metallo-β-lactamases (IMP) |  |  |
| Serine-carbapenemases (KPC) |  |  |
| Serine-carbapenemases (GES) |  |  |
| Others (specify) |  | |

- ***Pseudomonas aeruginosa***

| **RMs** | **Yes** | **No** |
| --- | --- | --- |
| Carbapenemases (OXA-48) |  |  |
| Metallo-β-lactamases (VIM) |  |  |
| Metallo-β-lactamases (IMP) |  |  |
| Metallo-β-lactamases (SPM) |  |  |
| Metallo-β-lactamases (GIM) |  |  |
| Serine carbapenemases (KPC) |  |  |
| Serine carbapenemases (GES) |  |  |
| Carbapenemases (OXA-40) |  |  |
| Carbapenemases (OXA-198) |  |  |
| Others (specify) |  | |

- ***Staphylococcus aureus***

| **RMs** | **Yes** | **No** |
| --- | --- | --- |
| *mecC* gene detection |  |  |
| Others (specify) |  | |

- ***Streptococcus agalactiae***

| **RMs** | **Yes** | **No** |
| --- | --- | --- |
| M phenotype |  |  |
| iMLSB phenotypes |  |  |
| cMLSB phenotypes |  |  |
| Others (specify) |  | |

- ***Streptococcus pyogenes***

| **RMs** | **Yes** | **No** |
| --- | --- | --- |
| M phenotype |  |  |
| iMLSB phenotypes |  |  |
| cMLSB phenotypes |  |  |
| Others (specify) |  | |

**If no, to which center do you send them?**

**Answer:**

**Do you select cases for this study (severe patient, clinician request, etc.) or do you do it in all cases?**

**Answer:**

10. **For the reported year (2023), can you indicate the profile and number of strains with multidrug resistance: MDR, XDR, PDR that you have detected? For which microorganisms?**

- YES*
- NO

*If yes, indicate the microorganisms:
