# Supplementary material for: The present and the future of antimicrobial resistance surveillance in Catalonia (Spain), 2024: from manual to automatic notification
Source: Antimicrob Resist Infect Control. 2026 Jun 30;15:125. doi: 10.1186/s13756-026-01781-0 (PMC13644157; doi:10.1186/s13756-026-01781-0)
Supplement: Supplementary file 3 [file 13756_2026_1781_MOESM3_ESM.docx]

**Supplementary text S2.** Survey on the surveillance of antimicrobial resistance in clinical microbiology laboratories (CMLs) in Catalonia (2023).

ANTIMICROBIAL RESISTANCE SURVEILLANCE IN CATALONIA

1. Healthcare centre data (Name of the centre, Responsible facultative, Email, Contact phone).

# 2. Phenotypic and genotypic techniques used for the study of AMR resistance (Select the techniques).

 Disk diffusion

 Microdilution (manual)

 Microdilution (automated)

 Gradient concentration strips (E-test (R))

 Immunochromatography (ICT)

 PCR

 MALDI-TOF

 Sequencing (1st Gen/Sanger)

 MLST

Sequencing (NGS/WGS)

# 3. Do you have Laboratory information system (LIS) in your centre?

 YES

NO

# 4. If yes, which LIS?
