# Supplementary material for: The present and the future of antimicrobial resistance surveillance in Catalonia (Spain), 2024: from manual to automatic notification
Source: Antimicrob Resist Infect Control. 2026 Jun 30;15:125. doi: 10.1186/s13756-026-01781-0 (PMC13644157; doi:10.1186/s13756-026-01781-0)
Supplement: Supplementary file 4 [file 13756_2026_1781_MOESM4_ESM.docx]

**Supplementary Table S1. Microorganisms, antibiotics and resistance mechanisms under surveillance in Catalonia.**

| **Microorganism** | **Antibiotics** | **Resistance mechanisms** |
| --- | --- | --- |
| *Acinetobacter baumannii* | Amikacin, ampicillin sulbactam, ciprofloxacin, colistin, imipenem and levofloxacin | OXA-23, OXA-40 and OXA-58; metallo-β-lactamases IMP, NDM and VIM |
| *Enterococcus faecalis* and *Enterococcus faecium* | Ampicillin, streptomycin, gentamicin and vancomycin | VanA and VanB phenotypes |
| *Escherichia coli* | Amikacin, ampicillin, amoxicillin/clavulanic acid, cefotaxime, ciprofloxacin, cotrimoxazole, ertapenem, gentamicin and imipenem | OXA-48; metallo-β-lactamases IMP, NDM and VIM; serine-carbapenemases GES and KPC |
| *Klebsiella pneumoniae* | Amikacin, amoxicillin/clavulanic acid, cefotaxime, ciprofloxacin, cotrimoxazole, ertapenem, gentamicin and imipenem | OXA-48; metallo-β-lactamases IMP, NDM and VIM; serine-carbapenemases GES and KPC |
| *Pseudomonas aeruginosa* | Amikacin, cefepime, ceftazidime, ciprofloxacin, colistin, imipenem, levofloxacin, meropenem, piperacillin/tazobactam and tobramycin | OXA-40 and OXA-198; metallo-β-lactamases GIM, IMP, SPM, NDM and VIM; serine-carbapenemases GES and KPC |
| *Staphylococcus aureus* meticillin sensible and resistant (MSSA and MRSA) | Benzylpenicillin, ciprofloxacin,  clindamycin, cotrimoxazole,  daptomycin, erythromycin,  gentamicin, levofloxacin, linezolid, oxacillin, rifampicin, tetracycline, tobramycin and vancomycin | *mecC* gene |
| *Streptococcus agalactiae* and *Streptococcus pyogenes* | Benzylpenicillin, erythromycin and clindamycin | M phenotype, iMLSB and cMSLB phenotypes |
