# Supplementary material for: The present and the future of antimicrobial resistance surveillance in Catalonia (Spain), 2024: from manual to automatic notification
Source: Antimicrob Resist Infect Control. 2026 Jun 30;15:125. doi: 10.1186/s13756-026-01781-0 (PMC13644157; doi:10.1186/s13756-026-01781-0)
Supplement: Supplementary file 5 [file 13756_2026_1781_MOESM5_ESM.docx]

**Supplementary table 3. Number of resistant antimicrobial susceptibility tests over total tests by microorganism and resistance type, 2016–2023 Catalonia (Spain)**

|  | **Number of resistent antimicrobial susceptibility tests/Number of total antimicrobial susceptibility tests (% of resistent tests)** | | | | | | | |
| --- | --- | --- | --- | --- | --- | --- | --- | --- |
| **Microorganism** | **2016** | **2017** | **2018** | **2019** | **2020** | **2021** | **2022** | **2023** |
| ***Acinetobacter baumannii* (CARB-R)** | 0/11  (0.00%) | 0/20  (0.00%) | 0/25  (0.00%) | 0/16  (0.00%) | 0/13  (0.00%) | 0/25  (0.00%) | 0/29  (0.00%) | 0/29  (0.00%) |
| ***Pseudomonas aeruginosa***  **(CARB-R)** | 449/2,073 (21.66%) | 402/1,771 (22.70%) | 267/1,403 (19.03%) | 278/1,511 (18.40%) | 383/2,166 (17.68%) | 422/2,581 (16.35%) | 411/2,730 (15.05%) | 348/2,697 (12.90%) |
| ***Escherichia coli***  **(CARB-R)** | 3/4,447 (0.07%) | 6/5,475 (0.11%) | 15/6,020 (0.25%) | 7/6,358 (0.11%) | 14/8,639 (0.16%) | 38/9,475 (0.40%) | 29/10,777 (0.27%) | 69/13,365 (0.52%) |
| ***Klebsiella pneumoniae* (CARB-R)** | 129/2,064 (6.25%) | 82/2,102 (3.90%) | 58/1,815 (3.20%) | 53/2,063 (2.57%) | 119/2,920 (4.08%) | 153/3,138 (4.88%) | 182/4,736 (3.84%) | 158/4,964 (3.18%) |
| ***Enterococcus faecalis***  **(VanR)** | 0/572  (0.00%) | 0/530  (0.00%) | 0/622  (0.00%) | 0/648  (0.00%) | 0/1,090 (0.00%) | 0/1,394 (0.00%) | 3/1,371  (0.22%) | 2/1,322  (0.15%) |
| ***Enterococcus faecium***  **(VanR)** | 1/367  (0.27%) | 0/366  (0.00%) | 1/383  (0.26%) | 0/460  (0.00%) | 8/752  (1.06%) | 17/919 (1.85%) | 3/1,071  (0.28%) | 22/956  (2.30%) |
| ***Escherichia coli***  **(3rd-gen. CPH-R)** | 355/2,471 (14.37%) | 392/2,858 (13.72%) | 464/3,034 (15.29%) | 511/3,335 (15.32%) | 762/4,808 (15.85%) | 963/5,931 (16.24%) | 1,123/6,592 (17.04%) | 1,142/6,404 (17.83%) |
| ***Klebsiella pneumoniae***  **(3rd-gen. CPH-R)** | 390/1,083 (36.01%) | 322/1,051 (30.64%) | 251/910 (27.58%) | 309/1,068 (28.93%) | 367/1,494 (24.56%) | 434/1,854 (23.41%) | 566/2,261 (25.03%) | 665/2,499 (26.61%) |
| **MRSA (Oxacillin)** | 204/616 (33.12%) | 243/685 (35.47%) | 246/715 (34.41%) | 305/808 (37.75%) | 299/1,284 (23.29%) | 301/1,500 (20.07%) | 405/1,712 (23.66%) | 411/1,992 (20.63%) |

3rd-gen. CPH-R, third-generation cephalosporin resistance (including cefotaxime); CARB-R, carbapenem resistance (including meropenem, ertapenem and imipenem); MRSA, methicillin-resistant *Staphylococcus aureus*; VanR, vancomycin resistance.
